# Traditional Chinese Medicine in nonalcoholic fatty liver disease: molecular insights and therapeutic perspectives

**DOI:** 10.1186/s13020-021-00469-4

**Published:** 2021-08-03

**Authors:** Xianmin Dai, Jiayi Feng, Yi Chen, Si Huang, Xiaofei Shi, Xia Liu, Yang Sun

**Affiliations:** Department of Clinical Pharmacy, Second Military Medical University/Naval Medical University, 200433 Shanghai, China

**Keywords:** TCM, NAFLD, Single herb, TCM compound, Mechanism

## Abstract

Nonalcoholic fatty liver disease (NAFLD) has become the world's largest chronic liver disease, while there is still no specific drug to treat NAFLD. Traditional Chinese Medicine (TCM) have been widely used in hepatic diseases for centuries in Asia, and TCM’s holistic concept and differentiation treatment of NAFLD show their advantages in the treatment of this complex metabolic disease. However, the multi-compounds and multi-targets are big obstacle for the study of TCM. Here, we summarize the pharmacological actions of active ingredients from frequently used single herbs in TCM compounds. The combined mechanism of herbs in TCM compounds are further discussed to explore their comprehensive effects on NAFLD. This article aims to summarize multiple functions and find the common ground for TCM treatment on NAFLD, thus providing enrichment to the scientific connotation of TCM theories and promotes the exploration of TCM therapies on NAFLD.

## Introduction

Nonalcoholic Fatty Liver Disease (NAFLD) is defined as the fatty liver with metabolic dysfunction. Recently, experts proposed Metabolic Associated Fatty Liver Disease (MAFLD) to replace NAFLD as a more appropriate overarching term [1, 2]. NAFLD manifests as fat storage syndrome accompanied by hepatocyte steatosis, ballooning degeneration, lobular inflammation and mostly fibrosis [3], which also determine the classification of NAFLD [4]. Traditionally, NAFLD comprises a wide spectrum, which includes nonalcoholic fatty liver (NAFL), nonalcoholic steatohepatitis (NASH), hepatic fibrosis (HF), hepatic sclerosis (HS) and even hepatocellular carcinoma (HCC) [5]. Taken together, nearly 1 billion people are affected globally, accompanied by a huge economic burden to society [6]. Moreover, an epidemiological investigation from US showed that the dramatically increase in liver mortality is related to the increased prevalence of NAFLD [7]. The multiple factors associated with higher risk of NAFLD including age, gender, hormonal status, ethnicity, diet, alcohol intake, smoking, genetic predisposition, the microbiota and metabolic status. These factors also contribute to NAFLD heterogeneity [6], which makes the treatment more difficult. Although several drugs have entered in phase 2 or 3 clinical trials, there is no approved drug therapy for NAFLD.

Traditional Chinese Medicine (TCM) have been widely used in hepatic diseases including NAFLD for centuries in Asia, and TCM’s holistic concept and differentiation treatment of NAFLD show their advantages in the treatment of this complex metabolic disease [8]. In TCM, the treatment of NAFLD mainly focused on the holism of hepatoprotection, which manifests as various forms and features in mechanism, including anti-oxidant stress, lipid metabolism modulation, anti-inflammation, anti-fibrosis and gut microbiota modulation. Thus, these pharmacological actions of active ingredients from frequently used single herbs in TCM compounds are summarized, such as Shan Zha (Hawthorn), Ze Xie (Rhizoma Alismatis), Fu Ling (Poria Cocos), Dan Shen (Radix Salvia Miltiorrhiza), Huang Qi (Radix Astragali), etc. The combined mechanism of herbs in TCM compounds are further discussed to explore their comprehensive effects on NAFLD. This review aims to summarize multiple functions and find the common ground for TCM treatment on NAFLD, and provides a comprehensive overview of TCM treatment strategies.

## The knowledge of NAFLD in TCM

The knowledge of NAFLD in TCM differs largely from that in Western medicine. In Western, the “Multiple Hit Theory” [9] is now widely accepted rather than the traditional “Two Hit Theory’’ [10]. The “Multiple Hit Theory” is based on the interaction between genetic and/or environmental factors, and changes in crosstalk between different organs and tissues. It involves a widespread metabolic dysfunction and multiple factors, including fat accumulation, insulin resistance, inflammation, gut-liver axis, dietary factors (fructose and sugar), oxidative stress, mitochondrial dysfunction, inflammation, endoplasmic reticulum stress, intestinal flora imbalance, and epigenetics [11]. Among them, liver fat accumulation, which may be caused by obesity and insulin resistance, still remains to be the “first hits” [9].

On the contrary, there is no corresponding nomenclature of NAFLD in TCM theory. While based on its symptoms and pathogenesis, NAFLD can be recognized as *hepatic syndromes like distention and fullness*, *phlegm syndrome like turbidity*, *hypochondriac pain*, *lump at the left hypochondrium* and *damp obstruction disease* [12]. According to the *Expert consensus on TCM diagnosis and treatment of nonalcoholic fatty liver disease (2017)*, NAFLD can be divided into five types: A. Congestion of dampness turbidity, B. Stagnation of liver-depression with spleen-deficiency, C. Accumulation knot of damp and hot, D. Syndrome of intermin-gled phlegm with blood stasis, E. Deficiency of spleen and kidney.

NAFLD is thought to locate mainly in the liver, closely related to lienal and renal functions [8]. Quantities of clinical and basic studies suggest that Stagnation of liver-depression with spleen-deficiency exists in most NAFLD patients [12], which is thought to be the main pathogenesis and always lead to earlier NAFLD. After that, damp obstruction, turbid phlegm, poor blood flow, and other syndromes occur together or alone in the latter stage, which further deteriorates NAFLD [8, 13].

## Pharmacotherapy in TCM treating NAFLD

In whether TCM or Western Medicine, lifestyle modification with healthy eating and regular exercise remain the first-line therapy in NAFLD [14]. However, it is not effective in all patients especially in those with decompensated disease or in advanced stage [15]. In Western medicine, although there is no approved drug therapy for NAFLD, some drugs are beneficial in treating NAFLD, such as insulin sensitizer, lipid-lowering drugs, antioxidants, weight-loss drugs and intestinal probiotics. These drugs aim to improve metabolic imbalance (hyperglycemia, hyperlipemia, etc.) and liver injury (fibrosis, etc.) occurred in NAFLD. Metformin and statins can reduce liver lipid deposition and promote material metabolism in patients with diabetes and hyperlipidemia [16, 17]. Pioglitazone and vitamin E may be beneficial in non-diabetic NASH patients, and pioglitazone is also proved to be effective on reversing NASH and improving fibrosis, which was recently confirmed in diabetic patients [18, 19]. As recent researches revealed more and more key targets involved in NAFLD, a variety of new drugs (PPAR agonists, FXR agonists, ASK inhibitors, etc.) are being explored to treat NAFLD.

In contrast, TCM achieved unique curative effects with the guiding concept of “holism” [20], and is proved to have a better effect on improving alanine aminotransferase level and liver steatosis in the treatment of NAFLD [21]. These TCM compounds used for hepatic diseases have been widely used in Asia for centuries with high safety. In China, some TCM compounds have been approved as Chinese patent medicine (Table 1) (Referred to http://drugs.dxy.cn. To 2021.6.30), while more TCM compounds are already in clinical trials as listed in Table 2. (Referred to http://www.chinadrugtrials.org.cn. To 2021.6.30).

**Table. 1:** Chinese patent medicine treating NAFLD

	Compounds	Prescriptions	Indications
1	Dang Fei Li Gan Capulse	Swertia diluta, Silybum Marianum	NAFLD
2	Huazhi Rougan Granule	Artemisia capillaris, Semen cassia, Rhubarb, Rhizoma alismatis, Porcine jelly, Hawthorn, Rhizoma atractylodis, Rhizoma Atractylodis Macrocephalae, Pericarpium trichosanthis, Fructus Ligustri, Fructus ligustri, Lycii, Cirsii Herba, Bupleurum, Liquorice	NAFLD
3	Shen Ze Shu Gan Caspulse	Hawthorn, Rhizoma Alismatis, Artemisiae Scopariae, Radix Astragali, Radix Puerariae, Radix Salvia Miltiorrhiza, Polygonum cuspidatum, Semen Cassiae, Rheum officinale, Radix Bupleuri	NAFLD
4	Ke Zhi Caspulse	Crustacean shell, Polygoni Multiflori Radix, Artemisiae Scopariae, Radix Salvia Miltiorrhiza, Radix Achyranthis Bidentatae	NAFLD
5	San Qi Zhi Gan Pills	Panax pseudo-ginseng, Cuscutae Semen, Curcumae rhizoma, Chrysanthemi Flos, Rhizoma Atractylodis Macrocephalae, Rhizoma Alismatis, Paeoniae Radix Alba, Lotus leaf, Citri Reticulatae Pericarpium Viride, Radix Paeoniae Alba, Hawthorn, Honey	Fatty liver
6	Qiang Gan Pills	Artemisiae Scopariae, Lsatidis Radix, Angelica, Paeoniae Radix, Radix Salvia Miltiorrhiza, Radix curcumae, Radix Astragali, Codonopsis Radix, Rhizoma Alismatis, Polygonati Rhizoma, Rehmanniae Radix, Dioscoreae Rhizoma, Hawthorn, Massa Medicata Fermentata, Gentianae Macrophyllae Radix, Radix Rhizoma Glycyrrhizae	Fatty liver
7	Qing Gan Jian Pi Granule	Hawthorn, Artemisiae Scopariae, Rhizoma Alismatis, Plantaginis Herba, Polygonum cuspidatum, Radix Salvia Miltiorrhiza, Rheum officinale, Semen Cassiae, Radix Astragali, Radix Bupleuri, Polygoni Multiflori Radix	Fatty liver
8	Da Huang Li Dan Pills	Rheum officinale, Rhizome of Conic Gymnadenia, Phmllanthi Fructus	Fatty liver
9	Silibinin Capsules	Silybum Marianum	Fatty liver

**Table. 2:** Registered clinical trials of TCM Compounds in China on NAFLD

Compounds	Register ID	Indications	Prescription	Phase	Status
Dan Shao Gan Kang Granules	CTR20140038	Nonalcoholic fatty liver (Stagnation of liver and spleen deficiency, Internal accumulation of damp-heat)	Radix Bupleuri, Angelica, Salvia miltiorrhiza, Radix Paeoniae Alba, Astragalus, Fructus Schisandrae schisandrae	Phase 2	Recruitment completed
Huazhi Rougan Granule	CTR20192578 CTR20160739	Nonalcoholic simple fatty liver with damp-heat syndrome	Artemisia capillaris, Semen cassia, Rhubarb, Rhizoma alismatis, Porcine jelly, Hawthorn, Rhizoma atractylodis, Rhizoma Atractylodis Macrocephalae, Pericarpium trichosanthis, Fructus Ligustri, Fructus ligustri, Lycii, Cirsii Herba, Bupleurum, Liquorice	Phase 4	Recruiting
Shuganzhi Tablets	CTR20180031	Nonalcoholic simple fatty liver (Stagnation of liver and spleen deficiency with damp-heat syndrome)	Bupleurum, Fructus aurantii, Radix curcumae, Seaweed, Atractylodes atractylodes rhizome, Poria cocos, Rhizoma alismatis, Safflower, Shenpanax ginseng, Hawthorn, Radix Astragali, Red Vine	Phase 2	Recruiting
Tiaozhi Qinggan Capsules	CTR20132184	Nonalcoholic simple fatty liver (Phlegm turbidity suppression)	Artemisia capillaris, Rhizoma alismatis, Hawthorn, Gardenia, Salvia miltiorrhiza, Radix curcumae, etc	Phase 2	Recruiting
Hedan Tablets	CTR20131131	Nonalcoholic fatty liver	Lotus leaf, Salvia miltiorrhiza, Hawthorn, Senna leaf, Fructus Psoraleae	Phase 2	Recruiting
Shuangqing Granule	CTR20130902	Nonalcoholic simple fatty liver	Gynostemma pentaphyllum, Salvia miltiorrhiza, Knotweed, Artemisia capillaris, Lotus leaf	Phase 3	Recruiting
Ganqing Granule	CTR20133003	Nonalcoholic simple fatty liver (Stagnation of phlegm and blood stasis)	Scutellaria barbata, North bean root, Artemisia capillaris, Ginseng, Acanthopanax, Radix Astragali	Phase 2	Completed
Danning Tablets	CTR20132381	Nonalcoholic simple fatty liver	Rhubarb, Knotweed, Citri Reticulatae Pericarpium Viride, White thatch, Tangerine peel, Radix curcumae, Hawthorn	Not Applicable	Completed
Jiangzhi Ninggan Capsule	CTR20131783	Nonalcoholic simple fatty liver (Stagnation of phlegm and blood stasis)	Rhizoma alismatis, Rhizoma atractylodis, Panax notoginseng, Magnolia officinalis, Radix Bupleuri, Artemisia capillaris, Hawthorn	Phase 2	Completed

Besides these ready-made medicine, TCM prescriptions emphasize on the importance of individualized therapy based on syndrome differentiation [22, 23]. For example, for the type of “Stagnation of liver-depression with spleen-deficiency”, Ge Gen Qin Lian Decoction (Macrocephalae Rhizoma, Pseudostellariae Radix, Radix Puerariae, Citrus reticulata Blanco, Carthami Flos, Pinelliae Rhizoma, Coptidis Rhizoma, Bambusae Caulis In Taenias, Scutellariae Radix, Pheretima), Chai Hu Decoction (Radix Bupleuri, Scutellariae Radix, Pinelliae Rhizoma, Codonopsis Radix, Radix Rhizoma Glycyrrhizae, Zingiberis Rhizoma Recens, Rhizoma Alismati, Macrocephalae Rhizoma, Poria Cocos, Hawthorn, Radix Salvia Miltiorrhiza, Jujubae Fructus) and Chai Hu Shu Gan Powder (Radix Bupleuri, Citrus reticulata Blanco, Chuanxiong Rhizoma, Nutgrass Galingale Rhizome, Fructus Aurantii, Paeoniae Radix Alba, Radix Rhizoma Glycyrrhizae) could effectively reduce the serum lipid levels and alleviate the fatty liver [24–26]. For other types, Huazhi Rougan Granule (in Table 2) are effective for the type of “Accumulation knot of damp and hot” [27]. Huazhuo Granule (Macrocephalae Rhizoma, Rhizoma Alismatis, Polyporus, Poria Cocos, Cmnamomi Mmulus) are used to treat NAFLD type of “Congestion of dampness turbidity” [28]. And for the type of “Syndrome of intermin-gled phlegm with blood stasis”, Qushi Huayu Decoction (Artemisiae Scopariae, Polygonum cuspidatum, Herba Hyperici Japonici, Turmeric, Gardenia jasminoides) is commonly used and proved to reduce ALT, AST as well as serum lipid levels in NAFLD patients [29].

Although the prescriptions differ from each other, Ding et al. tried to explore the medication rules of herbal prescriptions for NAFLD by analyzing randomized controlled trials of herbal prescriptions for treating NAFLD collected from CNKI, WanFang, VIP, SinoMed and PubMed databases. In the 88 prescriptions screened out, the commonly used herbs were listed in Table 3 [30]. As shown in Table 4, we also analyzed the frequently used herbs in Chinese patent medicine (Table 1) and ready-made TCM compounds in clinical trials (Table 2). Clarifying the effects and mechanisms of these herbs are fundamental to figure out the molecular insights and therapeutic perspectives of TCM on NAFLD, which is further discussed in the next section.

**Table. 3:** Frequently used herbs in TCM prescriptions in China on NAFLD [30]

	Single herb	Frequency
1	Hawthorn	60
2	Rhizoma Alismatis	54
3	Radix Salvia Miltiorrhiza	54
4	Radix Bupleuri	46
5	Semen Cassiae	41
6	Poria Cocos	40
7	Turmeric	36
8	Macrocephalae Rhizoma	35
9	Radix Rhizoma Glycyrrhizae	25
10	Citrus reticulata Blanco/Artemisiae Scopariae	24

**Table. 4:** Frequently used herbs in Chinese patent medicne and ready-made TCM compounds in clinical trials on NAFLD

	Single herb	Frequency
1	Hawthorn	11
2	Artemisiae Scopariae	10
3	Rhizoma Alismatis	9
4	Radix Salvia Miltiorrhiza	8
5	Radix Bupleuri	6
6	Rheum officinale	5
7	Radix Astragali	5
8	Polygonum cuspidatum	4
9	Macrocephalae Rhizoma	4
10	Semen Cassiae/Radix Rhizoma Glycyrrhizae/Citrus reticulata Blanco/Lotus leaf/Rhizoma atractylodis	3

## Effects and mechanisms of commonly used single herb drugs in TCM on NAFLD

Due to the complicated components in TCM compounds and herbs, the study of active components become the mainstream direction in the research of TCM. Here, we summarize the pharmacological effects of active ingredients from high-frequency single herbs in Tables 3 and 4. These herbs are classified according to the property and effect in TCM [31].

### Digestant drug

#### Shan Zha (Hawthorn)

Hawthorn is the dried ripe fruit of *Crataegus pinnatifida* Bge. var. Major N. E. Br. Or *Crataegus pinnatifida* Bge., which is usually used to improve digestion in TCM. Vitexin, quercetin, quercitin of flavonoids, maslinic acid, chlorogenic acid of organic acids, ursolic acid, and oleanolic acid of triterpenes are the main components of Hawthorn [31, 32]. Among them, vitexin, quercetin and maslinic acid are reported to intervene NAFLD.

Vitexin is a natural flavonoid compound with multiple pharmacological activities such as lipid metabolism modulation, anti-inflammation. Vitexin can not only suppress de novo lipogenesis but also enhance fatty acid oxidation and lipolysis, in addition, it can improve insulin signaling in HFD mice possibly through binding to leptin receptor and activating AMPK [33, 34]. Furthermore, vitexin could ameliorate chronic stress combined with NAFLD mice induced by HFD, through inhibiting TLR4/NF-κB signaling and the expression of proteins related to fatty acid synthesis [35].

Another component, quercetin can not only eliminate lipid droplets but also restore the upregulated total cholesterol and triglyceride levels in HepG2 cells co-cultured with high D-glucose and free fatty acid [36]. In rodent model, quercetin can prevent CdCl_2_-induced hepatic steatosis and fibrosis that through upregulating nuclear factor erythroid 2-related factor 2 [37]. Besides that, quercetin can revert gut microbiota imbalance and TLR-4 pathway induction mediated by endotoxemia, and then subsequently inhibit inflammasome response and reticulum stress pathway activation, leading to the blockage of lipid metabolism and gene expression deregulation, which helps to alleviate NAFLD in HFD mice [38]. Thus, quercetin exerts its protective effect on HFD-induced NAFLD development by means of integrative responses involving intestinal microbiota dysbiosis, related gut-liver axis activation and lipotoxicity blockage, subsequent inhibition of inflammasome response and reticulum stress pathway activation [39]. On the other hand, isoquercetin, which can enzymatically trans-glycosylated to quercetin, was also reported to suppress hepatic lipid accumulation by activating AMPK pathway and TGF-β signaling in HFD rat model with NAFLD [40].

Maslinic acid is a pentacyclic triterpenoid. In L02 cells treated with FFA, maslinic acid cannot reduce lipid accumulation through suppression of sterol element binding protein cleavage activating protein [41], but also decrease lipogenesis by activating AMPK in HepG2 cells. In mice with HFD-induced obesity, maslinic acid was reported to protect against hepatic steatosis through regulation of the Sirt1/AMPK signaling pathway [42, 43].

In addition, polyphenols from hawthorn peels shows a stronger protection against oxidative stress, which can not affect liver MDA level, activities of T-SOD and GSH-Px, but also regulate the expression of Nrf-2/ARE [44].

### Damp-clearing drugs

#### Ze Xie (Rhizoma Alismatis)

Rhizoma Alismatis, the dry rhizome of *Alisma orientalis* (Sam.) Juzep., is a kind of TCM with diuresis, lipid-lowering and dampness-removing effect. Alisol A, alisol B, acetate of alisol A, B, C, epialisol A, alismol and alismin are the main components of Rhizoma Alismatis [31, 32]. Alisol A and B have been proved to be effective on NAFLD [45].

In the human hepatic stellate cell line LX-2 cells modeled with MCD medium, Alisol A can regulate autophagy via the AMPK/mTOR/ULK1 pathway, it can also suppress reactive oxygen species (ROS) and inflammation in MCD mouse model [46]. Besides that, Alisol A cannot alleviate lipid and glucose metabolism, but also reduce hepatic steatosis and improve liver function in HFD mice [47]. In vitro, AA24A significantly reduces the number of lipid droplets, oil red O lipid content and triglyceride (TG) content in cells treated with palmitic acid [48]. Meanwhile, AA24A observably alleviates the level of blood lipids, glucose metabolism disorder and insulin resistance in HFD induced obese mice [47]. AA24A may activate AMPKα pathway through down-regulating SERBP-1c, ACC, Fas and up-regulating CPT1 and Acox1, thus effectively reduces hepatic steatosis and inhibits inflammation [48].

Alisol B can protect against MCD-induced NASH in mice via activating the FXR signaling pathway, thus decreasing the accumulation of lipids in the liver, hepatic lobular inflammation and pericellular fibrosis [47]. AB23A markedly attenuates the accumulation of TG and TC in liver and blood of HFD-Ovx treated ApoE-/- mice [49], and has a protective effect on liver injury and intrahepatic cholestasis induced by α-naphthyl isothiocyanate (ANIT) [50]. AB23A could activate pregnane X receptor (PXR) and farnesol X receptor (FXR), which play a key role in the metabolism of TG and TC [49]. Besides that, AB23A promotes liver regeneration after partial hepatectomy by up-regulating the expression of hepatocyte proliferation-related fork box M1B, cyclin B1 and cyclin D1, and AB23A attenuates liver injury by inhibiting CYP7a1 and inducing the expression of efflux transporter BSEP [45].

#### Yin Chen (Artemisiae Scopariae)

Artemisiae Scopariae was the the dry overground part of *Artemisia scoparia* Waldst.et Kit. or *Artemisia Capillaris* Thunb. Its main chemical active components are flavonoids like capillarisin, coumarins like scoparone, organic acids such as chlorogenic acid, and volatile oil [31, 32].

In macrophages, scoparone can alleviate lipopolysaccharide-induced immune responses partly by blocking TLR-4/NF-κB signaling, and regulate autophagy by inhibiting the ROS/P38/Nrf2 axis; and in an MCD diet-induced NASH murine model, it can improve hepatic steatosis, apoptosis, inflammation, and fibrosis [51, 52].

Chlorogenic acid can alleviate autophagy and insulin resistance by suppressing JNK pathway in rat model of NAFLD [53], and the combination of metformin and chlorogenic acid was found to be more effective at alleviating inflammation and lipid accumulation in HFD mice, which through increasing phosphorylation of AMP-activated protein kinase [54]. Chlorogenic acid combines with geniposide [55], caffeine [56] are also reported to have a positive effect on NAFLD. Furthermore, Altilix® supplement containing chlorogenic acid and luteolin improved hepatic and cardiometabolic parameters in subjects with metabolic syndrome in clinical research [57].

#### Hu Zhang (Polygonum cuspidatum)

**Fig. 1:**
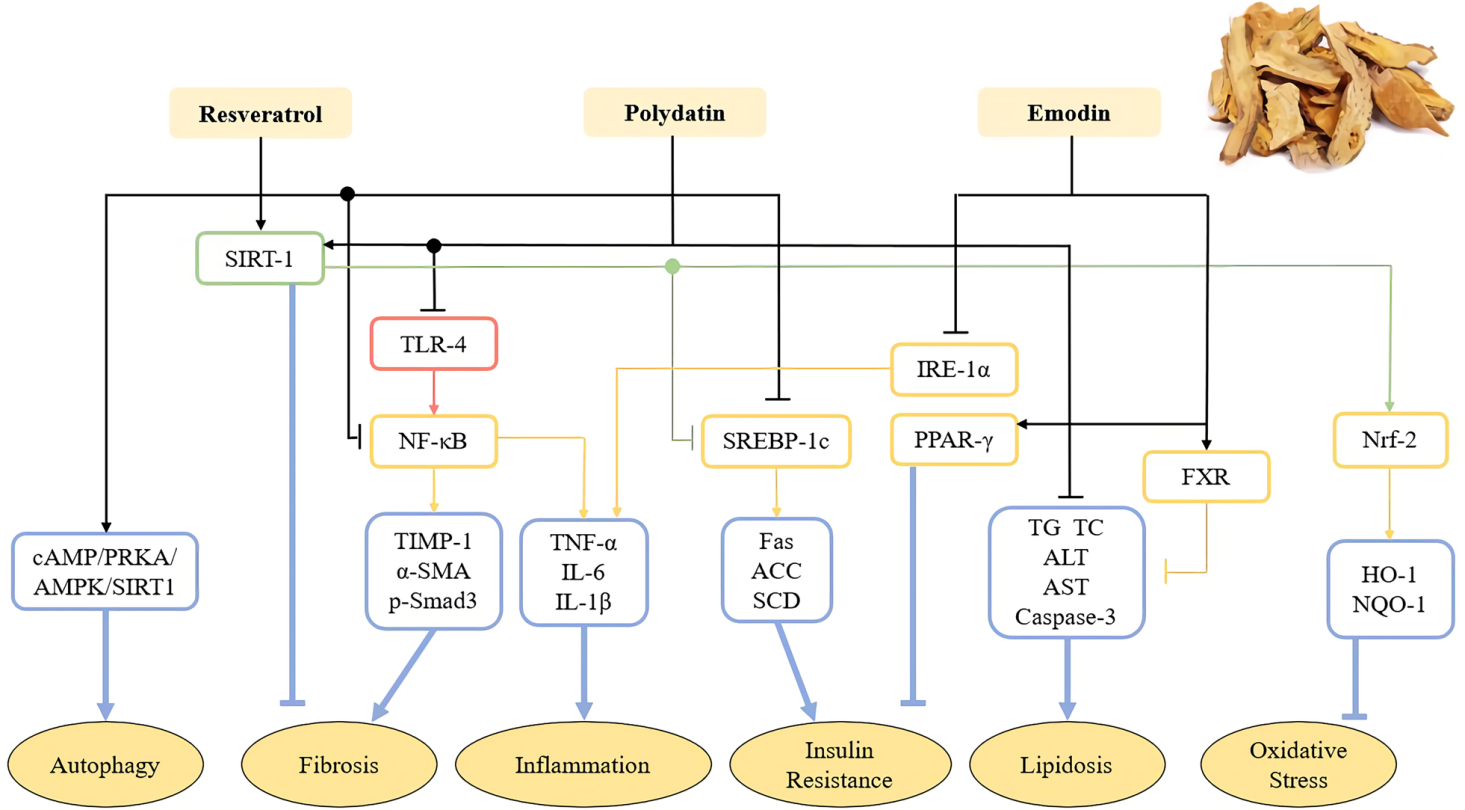
The effect and mechanism of Hu Zhang (Polygonum cuspidatum) on NAFLD. Resveratrol, polydatin and emodin are main active TCM monomers in Hu Zhang (Polygonum cuspidatum), influencing autophagy, fibrosis, inflammation, insulin resistance, lipidosis and oxidative stress.They work together to exert a therapeutic effect on NAFLD.

*Polygonum cuspidatum* is the dry rhizome and root of *Polygonum cuspidatum* Sieb. Et Zucc, which can protect against liver/gallbladder injury and have multiple effects such as dispelling jaundice, clearing heat and detoxification, promoting blood circulation and removing blood stasis, dispelling wind and dampness, resolving phlegm and relieving cough in TCM [58]. The active ingredients include resveratrol, emodin of anthraquinones, quercetin, polydatin and its derivatives of flavonols, coumarin and lignan [59]. Resveratrol, polydatin and emodin are main active components and work together to exert a therapeutic effect on NAFLD (Fig. 1).

Resveratrol has the effects of anti-inflammation, anti-oxidative stress, anti-fibrosis, alleviating insulin resistance and regulating autophagy. It has been reported that resveratrol can reduce the levels of pro-inflammatory cytokines TNF-α, IL-6 and IL-1 β and inhibit inflammation in HFD mice through NF-κB pathway. Resveratrol can markedly increase the activity of SIRT1 and partly induce autophagy through cAMP-PRKA-AMPK-SIRT1 signal pathway to improve NAFLD [60]. Resveratrol can also regulate the expression of Fas and SREBP-1c, SCD, ACC, and reduce triglycerides and insulin resistance in fructose-induced NAFLD through activating SIRT1 [61].

Polydatin is the glycoside form of resveratrol, which is the most abundant form of resveratrol in nature [62]. Polydatin relieves NAFLD by inhibiting inflammation, anti-fibrosis, anti-oxidative stress and regulating liver fat. It is reported that polydatin cannot reduce serum total cholesterol (TC), triglyceride (TG), ALT, AST, caspase-3, but also alleviate liver fat accumulation [59]. The expression of SIRT-1 increases in hepatocytes treated by polydatin, resulting in the increasing of gene Nrf2 nuclear translocation, and the increased expression of downstream target proteins HO-1 and NQO1, ultimately inhibits oxidative stress [63]. Polydatin also significantly downregulates the expression of TLR4, and reverses the increase the mRNA levels of TNF-α, IL-6 and IL-1β, thus inhibits inflammation in MCD mice. Additionally, polydatin relieves liver fibrosis through downregulating the expression levels of TIMP-1, α-SMA and the phosphorylation of Smad-3 [64].

Emodin is a natural anthraquinone derivative with a wide range of pharmacological activities. It has been reported that emodin can block endoplasmic reticulum stress conduction of IRE1α and its downstream molecules, and reduce the levels of TNF-a, IL6, IL-1β, which plays an anti-inflammatory effect on NAFLD. Meanwhile emodin can reduce insulin resistance and improve diabetes by regulating PPAR-γ pathway [65]. Moreover, emodin also reduces blood lipid levels, and improves obesity and lipid deposition by activating FXR signaling pathway [66].

### Blood-activating and stasis-eliminating drugs

#### Fu Lin (Poria Cocos)

Poria is the dried sclerotium of *Poria cocos* (Schw.) Wolf, which has the effect of diuresis and detumescence, spleen invigorating and stomach protection, nourishing and sedative. The main components are pachyman, pachymic acid, proteins, ergosterols and inorganic salts [31, 32]. Triterpenes and polysaccharides are the ingredients which have the pharmacological effects include regulating immunity, anti-inflammation, anti-oxidation, anti-tumor, liver protection and etc. [67].

Triterpenes mainly show obvious anti-inflammatory activities, as well as other pharmacological effects such as anticancer and hypoglycemic. Poria Cocos terpenoids can promote adipocyte differentiation in vitro while reduce blood glucose as insulin sensitizers in vivo [68].

Poria cocos polysaccharides mainly show immunomodulatory, anti-inflammatory, hepatoprotective activities, and other pharmacological effects such as anti-cancer, antioxidant stress [67, 69]. Poria Cocos polysaccharides markedly downregulate the level of ALT, LD, TNF-α and IL-6 in serum of mice with liver injury induced by acetaminophen, inhibit inflammatory cells infiltration and apoptosis in liver tissue, so as to alleviate liver injury [67].

#### Dan Shen (Radix Salvia Miltiorrhiza)

Radix salvia miltiorrhiza is the dried root and rhizome of *Salvia miltiorrhiza* Bge*.*, which was used to treat liver disease for century. The ingredients of radix salvia miltiorrhiza are divided into two categories, water-soluble and fat-soluble. The former includes phenolic acids, such as tanshinic acid A, B, C and Salvianolic acid A-I. The fat-soluble components are mainly diterpene quinones include tanshinones and rolitazones [31, 32].

Salvianolic acids are among the most efficacious polyphenol compounds extracted from Radix Salvia miltiorrhiza. In vitro, salvianolic acid A pre-treatment protects against palmitic acid-induced hepatocyte cell death in HepG2 cell [70]. In the same research, salvianolic acid A administration alleviates hepatic steatosis and liver injury in high-fat and high-carbohydrate diet-fed mice. In addition, salvianolic acid A protects against HFD-induced NAFLD by ameliorating both hepatic lipid accumulation and inflammation, and the anti-inflammatory effects may partially due to regulation of the TXNIP/NLRP3 pathways [71].

Salvianolic acid B can alleviate NASH by protecting the morphological characteristics and functions of liver mitochondria, regulating lipid metabolism, oxidative stress, lipid peroxidation and inhibiting apoptosis in rats [72, 73]. It can also regulate SIRT1-mediated HMGB1 deacetylation to protect against HFD or PA induced hepatic steatosis and inflammation [74]. In another study, it is reported to regulate the multiple targets such as PPARα, CYP1A2, and MMP2 to exert lipid metabolism modulating effect, antioxidant effect and anti-fibrogenesis effect [75].

#### Yu Jin (Curcumae Radix)

Curcumae Radix is the dried tuberous root of *Curcuma wenyujin* Y. H. Chen et C. Ling, *Curcuma. Longa* L., *Curcuma. Kwangsiensis* S. G. Lee et C. F. Liang or *Curcuma. Phaeocaulis* Val., which is a spice and medicinal herb widely used in TCM. Curcumin, turmerone, volatile oils, curcumol, and other starch, polysaccharides, fatty oils, rubber, phellandrene are the main chemical components [31, 32]. Curcumin and curcumol are two primary bioactive compounds, which both exhibit multiple biological activities on NAFLD, such as anti-oxidant stress, lipid metabolism modulation, anti-inflammation, etc.

Curcumin can reduce hepatic succinate accumulation and prevented stellate cell activation via blocking succinate/HIF-1α signaling, which help to prevent liver fibrosis in mouse primary hepatic stellate cells [76]. In an animal model, curcumin attenuates the hepatic steatosis in HFHFr-fed mice through regulating endogenous and exogenous metabolism via Nrf2-FXR-LXR pathway to control lipid synthesis [77], and ameliorates liver injury via upregulating anti-oxidative responses by the action of GSH-Px and SOD in NAFLD rats [78]. Moreover, curcumin affects the abundance of several representative families in gut microbial communities, including prevotellaceae, bacteroidaceae, and rikenellaceae, and alleviates hepatic steatosis in part through stain-specific impacts on hepatic steatosis associated phylotypes of gut microbiota in rodent models [79, 80]. In clinical trial, supplementation with curcumin containing phosphatidylserine and piperine could improve glycemic factors, hepatic function and serum cortisol levels in subjects with overweight and impaired fasting glucose [81]. While characterizing the serum metabolic profile of the patients with NAFLD at the intervention of curcumin, the results indicated that the targets of it included some amino acids, TCA cycle, bile acids, and gut microbiota [82].

Curcumol can not only suppresse hepatic stellate cells proliferation and activation but also ameliorate the carbon tetrachloride (CCl4)-induced mice liver fibrosis, which was associated with regulating hepatic stellate cells necroptosis through increasing the phosphorylation of receptor-interacting protein kinase 1 (RIPK1) and receptor-interacting protein kinase 3 (RIPK3) [83].

### Tonic drugs

#### Huang Qi (Radix Astragali)

**Fig. 2:**
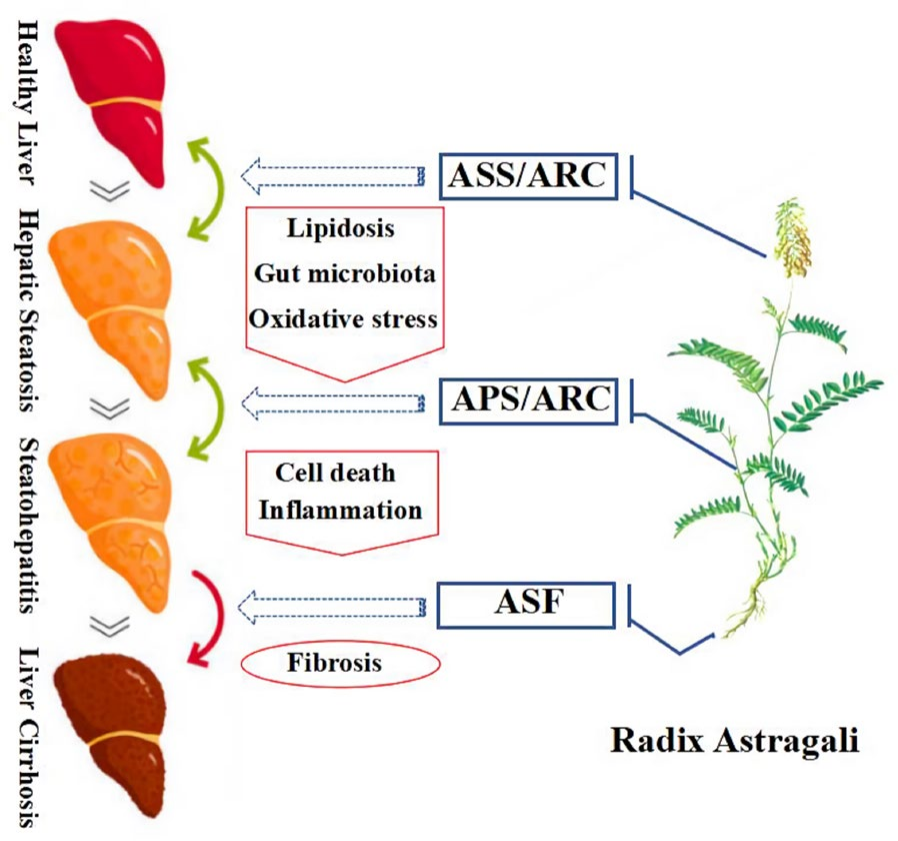
The value of Radix Astragali in NAFLD. The active monomer or effective constituents of radix astragali effect the different process of NAFLD. ASS: Astragaloside; ARC: Astragali Radix related compounds; APS: Astragalus Polysaccharides; ASF: Astragalus Flavone

Radix Astragali is the dried root of *Astragalus membranceus* (Fisch.) Bge. or *Astragalus membranceus* (Fisch.) Bge*. var.. Mongholicus* (Bge.) Hsiao. The components of Radix Astragali include flavonoids, triterpenes (astragaloside I-IV), polysaccharides, and others alkaloids, glucuronic acid, various trace elements. Among them, the main ingredients are astragalus polysaccharide, astragaloside IV, astragalus flavone, etc. [31, 32]. Radix Astragali can reduce liver lipid deposition [84], improve inflammation [85], inhibit liver fibrosis [86] and protect liver injury. Thus, Radix Astragali have therapeutic effects on all stages of NAFLD (Fig. 2).

Astragalus polysaccharides (APS) include heteropolysaccharides and glucans. In vitro, APS can promote glucose uptake and increase insulin sensitivity in 3T3-L1 adipocytes, which may involve the miR-712-PPAR-γ-PI3K/AKT-GLUT4 signaling pathway [87]. APS can also improve insulin resistance in NASH rats through the phosphorylation of IRS-1 and upregulating of ACE2, Mas and Ang-(1–7) [88]. Moreover, it can regulate the glucose and lipid metabolism in NAFLD rats, as well as the serum AST, ALT [89, 90].

Astragalus saponins (ASS) mainly includes five major saponins, astragalosides I, II, and IV, and isoastragaloside I and II. Astragalosides IV is the qualitative control biomarker of Radix Astragali. In vitro, Astragalosides IV attenuates free fatty acid-induced ER stress and lipid accumulation in hepatocytes via AMPK activation [91]. In a rodent model, it can improve lipid metabolism in obese mice by alleviation of leptin resistance and regulation of thermogenic network. Moreover, it can inhibit adipose lipolysis and reduce hepatic glucose production via Akt dependent PDE3B expression in HFD mice [92, 93].

Astragalus flavonoids (ASF) include β-sitosterol, formononetin, calycosin, and glucoside and so on. Calycosin combined puerarin could improve insulin resistance by regulating glucose and lipid metabolism [94]. Total flavonoids of astragalus could inhibit liver fibrosis in CCl_4_-induced rats, its potential mechanism is related to TGF-β1/Smad signaling pathway in inflammatory response [86]. In a conclusion, astragalus flavones could affect glucose and lipid metabolism, insulin resistance, improve liver fibrosis and prevent NASH.

Astragali Radix related compounds include Qiyin granules and Huangqi powder. Qiyin granules (Radix Astragali, Herba Artemisiae Scopariae, Folium Ilecis Latifoliae) can improve the syndrome of NAFLD in TCM (liver depression and spleen deficiency), liver ultrasound and blood lipid [95]. Huangqi powder can regulate glucose and lipid metabolism, protect liver and reduce body weight [96].

#### Gan Cao (Radix Rhizoma Glycyrrhizae)

Radix Rhizoma Glycyrrhizae, also named licorice, is the dried root and rhizome originated from *Glycyrrhiza uralensis* Fisch., *Glycyrrhiza inflata* Bat. or *Glycyrrhiza glabra* L. Licorice mainly contains triterpenoid saponins (glycyrrhizin, etc.), flavonoids, coumarins, polysaccharides, alkaloids and amino acids [31, 32]. Licorice flavonoids and glycyrrhizin are active ingredients, which can improve carbohydrate, lipid metabolic disorders, and insulin resistance [97]. It is reported that licorice root extract significantly decreased ALT and AST levels in NAFLD patients in a randomized controlled clinical trial [98].

In vitro, glycyrrhizin was found to stabilize lysosomal membranes, inhibit cathepsin B expression and enzyme activity, inhibit mitochondrial cytochrome c release, and reduce FFA-induced oxidative stress. In rodent models, glycyrrhizin can dampen the activation of NLPR3 inflammasome and restore bile acids homeostasis [99]. It also potently inhibit MCD diet-induced liver lipids accumulation, inflammation, and fibrosis [100]. Moreover, glycyrrhizin could metabolize into glycyrrhetinic acid, which is a novel AKR1B10 inhibitor. Glycyrrhetinic acid further restores the balance of retinol metabolism, then resolve the fatty and inflammatory lesions in liver of NAFLD/NASH mice [101].

#### Bai Zhu (Atractylodes macrocephala Koidz)

Atractylodes macrocephala is the rhizome of the plant *Atractylodes macrocephala* Koidz. The active components include volatile oil, atractylodes ketone, atractylodes alcohol, atractylodes ether, juniper, atractylodes lipids, and other fructose, glycans, atractylodes macrocephala polysaccharides, amino acids, and vitamin A [31, 32]. The most significant active substances in its extract are atractylenolides and polysaccharides, which have anti-inflammatory, antioxidant stress, improving fat and energy metabolism, liver protection and other pharmacological effects.

Atractylenolides has a predominant anti-inflammatory activity. Atractylenolides I can inhibit the production of NO, TNF-α, IL-1β, IL-6, vascular endothelial growth factor and placental growth factor in the mouse air punch model induced by Freund's complete adjuvant as well as in the mice peritoneal macrophages induced by lipopolysaccharide. Other sesquiterpenes also showed anti-inflammatory activities [102].

Atractylodes macrocephala polysaccharides play an important role in heat stress, immunomodulation and anti-inflammation. Polysaccharides can alleviate diet-induced liver injury by regulating the activities of antioxidant enzymes and liver lipid metabolism [103]. It has been reported that polysaccharides markedly inhibit the levels of IL-1β, IL-6 and TNF-α that are induced by LPS and increase the level of serum IL-4 in mice [102]. In addition, polysaccharides can also downregulate the levels of CAT, GSH-Px, SOD, iNOS and MDA in serum. More importantly, polysaccharides alleviate the damage of hepatocytes induced by LPS by upregulating the expression of TLR4, MyD88, IkB-α and NF-κB, as well as upregulating the expression of TLR4-MyD88-NFB [104].

### Antipyretic drugs

#### Jue Mingzi (Semen Cassiae)

Semen Cassiae is the dried and mature seed of *Cassia obtusifolia* L. or *Cassia tora* L., which is a well-known medicinal food in China and is used to clear liver heat, sharpen vision, lubricate the intestines, and promote bowel movement. It contains rhein, emodin, aloe emodin, cassia, orange in of anthraquinones, and cassiaside, cassioside, cassia lactone of naphthopyrrolidones, and other sterols, fatty acids, sugars and proteins [31, 32].

Rhein can ameliorate fatty liver disease through energy balance, hepatic lipogenic regulation, and immunomodulation in HFD induced obese mice [105]. Rhein can reduce the expression of fat mass and obesity-associated protein [106], and also mitigate oxidative stress and lipid metabolism in HFD rats [107], which may be associated with downregulation of TLR4, MYD88 and Cyr61 [108]. Rhein lysinate can also improve hepatic function through decreasing hepatic adipose infiltration and the expression of inflammatory factors in diabetic mouse model induced by STZ and diabetic food [109].

Emodin have an effect of hepatoprotection, lipid metabolism regulation and anti-inflammation in liver, which has been fully illustrated in in previous part of *Polygonum cuspidatum*.

Cassia glycosides can protect against *tert*-butylhydroperoxide induced cell death in HepG2 cells, and this hepatoprotective effects were exerted through nuclear factor erythroid-2-related factor 2 (Nrf2)-dependent antioxidative signaling [110, 111]. In high fat and high sugar model of NAFLD, cassia glycosides can obviously improve liver function and regulate blood lipids when compared with the commonly used drug polyene phosphatidylcholine in clinic, which is related with the inhibition of SREBP-1c expression in liver [112]. Furthermore, Cassia glycosides can improve the inflammatory in liver of HFD rats through regulation of TLR4 and NF-κB [113].

#### Shui Feiji (Silybum Marianum)

Silybum Marianum is the herb of *Silybum marianum* (L.) Gaertn, the extra of which are widely used as a complementary and alternative treatment of various hepatic conditions, the active components mainly include silybin and silymarin.

Silybin is used as a hepatoprotective agent in NAFLD therapy in TCM. In vitro, silybin can affect lipogenic pathways, and reduce cell viability in cultured hepatocytes exposed to fructose and fatty acids [114]. In NAFLD mice with HFD, silybin can not only reverse metabolic disorders caused by high fat diet feeding, but also regulate hepatic lipid accumulation and metabolic pathways [115], which may through the NAD + /SIRT2 pathway [116]. In clinical trial, the proportion of NAFLD patients treated with silybin combined with vitamin A and D shows a statistically significant improvement in metabolic markers, oxidative stress, endothelial dysfunction [97, 117].

Silymarin reduced ALT, hepatic inflammation, oxidative stress, and apoptosis both in on fat-laden human hepatocytes and in juvenile NASH mice [118]. Silymarin can also attenuate hepatic steatosis in HFD mice through regulation of lipid metabolism and oxidative stress, which is benefit to the circulation system [119]. In addition, silymarin can suppress the activation of HSCs and increase NRF2 translocation in MCD diet induced NASH [120, 121]. In a randomized trial of silymarin to treat NASH, silymarin can reduce liver fibrosis and it appears to be safe and well tolerated. However, the result remains to be confirmed in a larger trial (ClinicalTrials.gov: NCT02006498).

### Relieving drug

#### Chai Hu (Radix Bupleuri)

Radix Bupleuri is the dried root of *Bupleurum chinense* DC. or *Bupleurum scorzo-neri folium* Willd. It is commonly used as a hepatoprotectant in TCM, which includes variety of chemical constituents, such as saikosaponins, volatile oils, flavonoids, sterols and polysaccharides [31, 32]. Saikosaponin A, and quercetin are two components that both can intervene the progression of NAFLD.

Saikosaponin A is a triterpene saponin derived from Radix bupleuri. Saikosaponin A can not effectively inhibit liver steatosis but also restore liver lipid metabolism and liver function in HFD mice, which may be related to insulin resistance [122].

Quercetin existed in many TCMs like Radix Bupleuri, Semen Cassiae, Hawthorn and Mulberry leaf. There are reports shown that quercetin can regulate lipid metabolism, gut microbiota and anti-inflammation, which has been fully described in previous part of Semen Cassiae.

### Purgating drug

#### Da Huang (Rheum officinale)

Rheum officinale is the dried root and rhizome of *Rheum palmatum*, L., *Rheum tanguticum Maxim. ex* Balf. or *Rheumoj-flcinale* Baill. Rheum officinale mainly contains anthraquinones derivatives and anthrones [31, 32]. Anthrones are the reduction products of anthraquinones. Anthraquinones can be divided into free aglycone and binding aglycone. Binding aglycone is free aglycone conjugating with glucose, mainly includes various emodin glycosides. Although the content of free aglycone is low, its activity is highly, such as rhein, chrysophanol, emodin, aloe-emodin and physcin.

Chrysophanol can attenuate NAFLD in neonatal rats fed with HFD via regulating lipid synthesis, lipidolysis and inflammation [123]. Rhein and its derivative emodin play a positive role in the treatment of NAFLD, which has been fully referred in previous part of Rheum officinale.

### Others

Besides these high-frequency herbs used in TCM compound listed in Table 3 and Table 4, some TCM monomers also exhibit their potential in treating NAFLD.

Berberine is an isoquinoline alkaloid, of which the reports on NAFLD are abundant and suggest that berberine is a promising candidate for treatment on NAFLD. Berberine can be isolated from Huang Lian (Coptidis Rhizoma), Huang Qin (Baical Skullcap Root), San Ke Zhen (Barberry Root) and many other herbs. More than a hundred herbs are proved to contain berberine and Huang Lian (Coptidis Rhizoma) is the most famous one among them. Berberine can significantly decrease lipid accumulation, ameliorated reactive oxygen species (ROS) and lipid peroxides, TNFα, and phosphorylation of NF-κB p65 both in MCD mice and in AML12 cells induced by MCD/LPS or PA [124]. In addition, berberine can also regulate liver TG synthesis and hepatic steatosis through the activation of AMPK-SREBP-1c-SCD1 pathway both in HFD mice and in HepG2/ AML12 cells exposed to high glucose and palmitic acid [125]. In conclusion, berberine can not only reduce hepatic lipid accumulation by modulating fatty acid synthesis and metabolism, but also restore the bile acid homeostasis [126]. Furthermore, it markedly inhibits inflammation by reducing immune cell infiltration, neutrophil activation and inflammatory gene expression. It also inhibits hepatic fibrosis by modulating the expression of multiple genes involved in hepatic stellate cell activation and cholangiocyte proliferation. In clinical trial, berberine can induce a substantially greater change in serum lipid species compared with mere lifestyle intervention after treatment, which ameliorates NAFLD and related metabolic disorders [127, 128]. ClinicalTrials.gov: NCT03198572.

Baicalin and wogonin are the primary active compounds originating from Huang Qin (Baical Skullcap Root), which is the dried root of the perennial herb *Scutellaria baicalensis* Georgi. Baicalin cannot reduce pyroptosis by blocking NLR pyrin domain containing 3–gasdermin D signaling in hepatocyte induced with free fatty acids [129], but also alleviate palmitic acid-induced cytotoxicity in AML12 cells via suppression of ER stress and TXNIP/NLRP3 inflammasome activation [130]. In rodent models, baicalin can effectively protect mice against MCD induced NAFLD/NASH [131]. In addition, baicalin is also involved in the interactions of the liver-gut axis by regulating TGR5, FXR, bile acids and the microbiota, which regulates intestinal flora by promoting the production of SCFAs [132].

On the other hand, wogonin can attenuate liver fibrosis via regulating the activation and apoptosis of hepatic stellate cells [133], palmitate-induced oxidative stress and inflammatory response in NCTC1469 cells [134]. In rodent model, wogonin supplementation significantly improved metabolic parameters in NAFLD mice, including body weight, blood glucose, insulin resistance, adiponectin, blood lipids, aminotransferases and hepatic histopathology [134].

## Combined effects of compounds used in TCM on NAFLD

TCM compounds are characterized by the concept of holism and differentiation treatment. With a multi-ingredient and multi-target-pathway pharmacological action, TCM compounds are compatible with the complex pathogenesis of NAFLD to mitigate it [8].

### Commonly used drug combinations in prescription

**Fig. 3:**
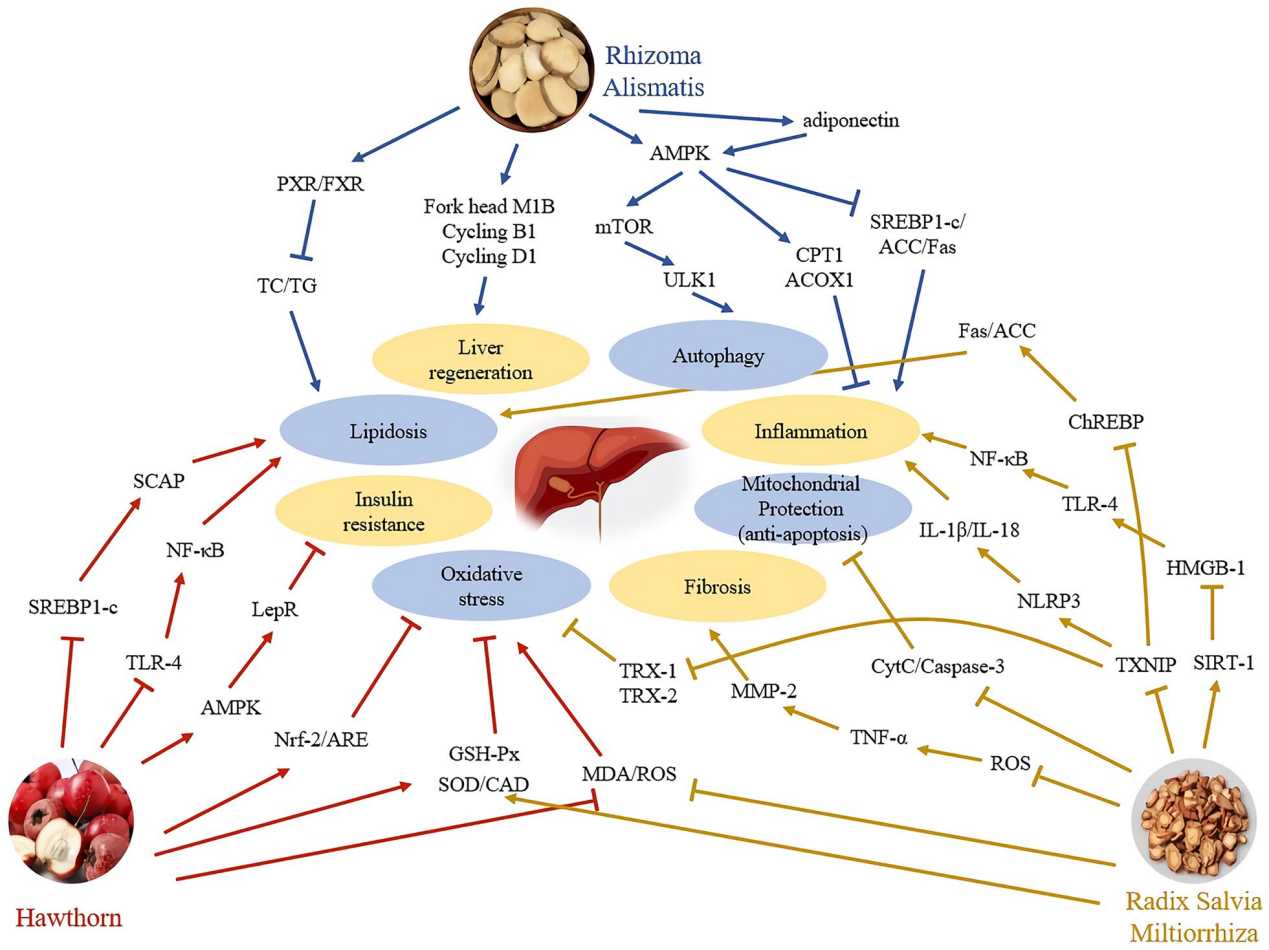
The effect and mechanism of commonly used drug combinations in prescription on NAFLD. “Hawthorn-Rhizoma Alismatis”, “Rhizoma Alismatis-Radix Salvia Miltiorrhiza” and “Hawthorn-Radix Salvia Miltiorrhiza” are top 3 drug combinations in herbal prescriptions. They exerts a more comprehensive effect on treating NAFLD than single herb

In the analysis of 88 herbal prescriptions screened out by Ding et al. collected from databases, the top 3 drug combinations are “Hawthorn-Rhizoma Alismatis”, “Rhizoma Alismatis-Radix Salvia Miltiorrhiza” and “Hawthorn-Radix Salvia Miltiorrhiza”, with a high frequency of over 40 in 88 prescriptions [30]. The digestant drug Shan Zha (Hawthorn) mainly improve lipid metabolism and insulin resistance, and protect against oxidative stress in NAFLD. The damp-clearing drug Ze Xie (Rhizoma Alismatis) could alleviate lipidosis as well. Besides, it also has effects on liver regeneration, inflammation and autophagy, which supplement to the effect of Hawthorn. On the other hand, the blood-activating and stasis-eliminating drug Dan Shen (Radix Salvia Miltiorrhiza) have more effects on lipid metabolism, oxidative stress, inflammation, cell death and fibrosis. These are complementary to the effects of Hawthorn and/or Rhizoma Alismatis. As shown in Fig. 3, the combinations of three herbs play a more comprehensive role in treating NAFLD than single herb.

### Chinese patent medicine

**Fig. 4:**
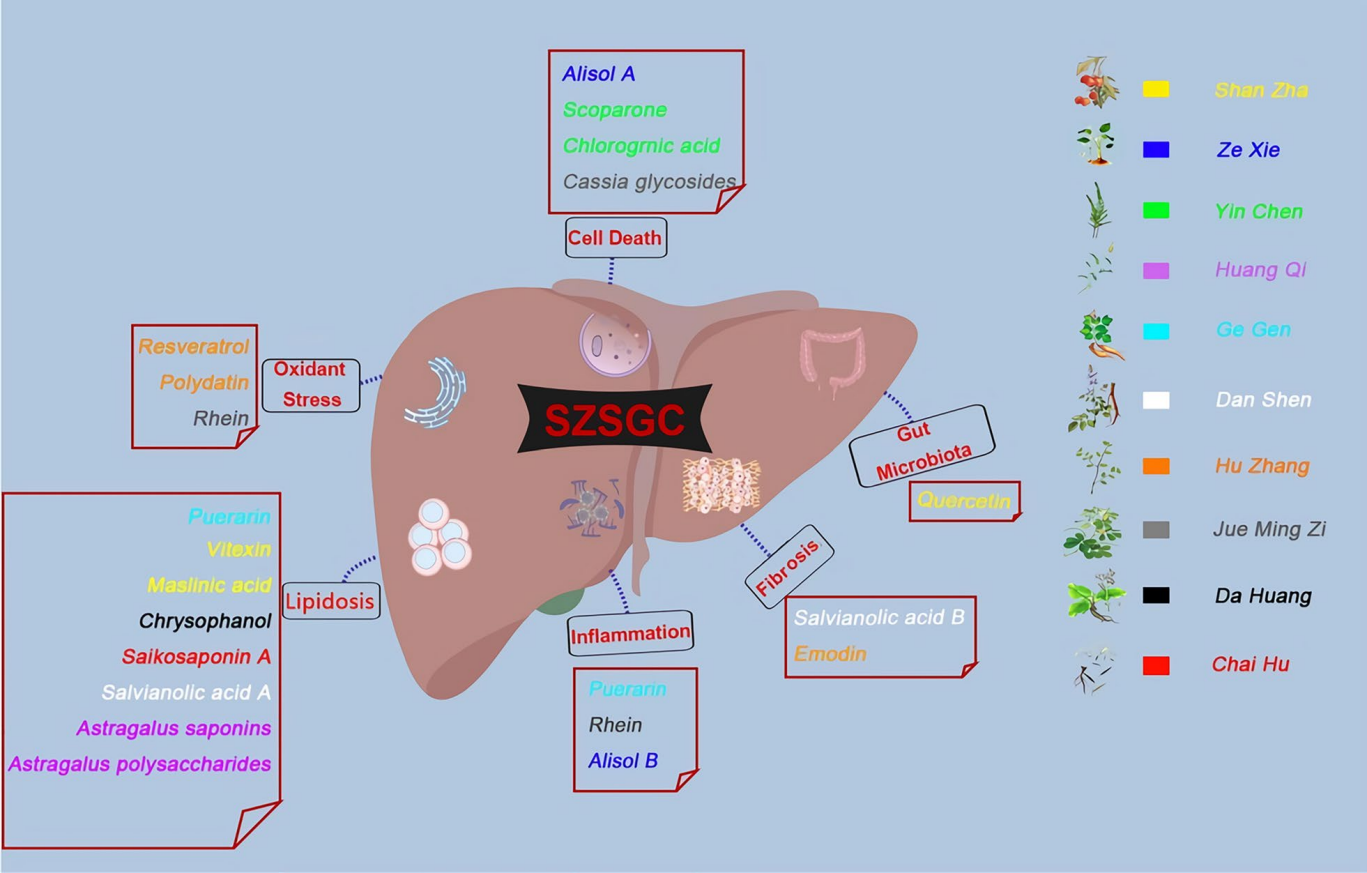
The integrated effect of Shen Ze Shu Gan Caspulse(SZSGC) on NAFLD. Shen Ze Shu Gan Caspulse are consisted of Shan Zha (Hawthorn), Ze Xie (Rhizoma Alismatis), Yin Chen (Artemisiae Scopariae), Huang Qi (Radix Astragali), Ge Gen (Radix Puerariae), Dan Shen (Radix Salvia Miltiorrhiza), Hu Zhang (Polygonum cuspidatum), Jue Ming Zi (Semen Cassiae), Da Huang (Rheum officinale) and Chai Hu (Radix Bupleuri). and all of these herbs are presented in different color. The active components was shown in the same color as their single herb origin. All active components exert a integrated effect on NAFLD, including oxidant stress, cell death, lipid metabolism, inflammation, fibrosis, gut dysbiosis, etc.

Besides individualized prescriptions, some ready-made TCM compounds have been patented to treat NAFLD in China (Table 1), while some others are in clinical trials (Table 2). These TCM compounds are made into fixed formulas, which are proved to be effective on NAFLD treatment. Although these Chinese patent medicine could not meet the need of differentiation treatment, they still have the character of TCM “holism”. For example, Shen Ze Shu Gan Caspulse are consisted of Shan Zha (Hawthorn), Ze Xie (Rhizoma Alismatis), Yin Chen (Artemisiae Scopariae), Huang Qi (Radix Astragali), Ge Gen (Radix Puerariae), Dan Shen (Radix Salvia Miltiorrhiza), Hu Zhang (Polygonum cuspidatum), Jue Ming Zi (Semen Cassiae), Da Huang (Rheum officinale) and Chai Hu (Radix Bupleuri). Each single herb may have more than one active components affecting NAFLD. All of these herbs have been discussed above except for Radix Puerariae, whose major active component is puerarin. Puerarin exerts a regulatory effect on lipid accumulation by decreasing lipogenesis in hepatocytes [135, 136]. Overall, Shen Ze Shu Gan Caspulse exert a integrated effect on NAFLD, including oxidant stress, cell death, lipid metabolism, inflammation, fibrosis, gut dysbiosis, etc. (Fig. 4).

## Conclusions and perspectives

NAFLD is a complicated metabolic disorder featured mainly by hepatic steatosis, inflammation and fibrosis. At early stages (simple liver steatosis and early liver inflammation), patients can restore liver function by exercise and diet changes. However, as the disease progresses, drug intervention or even liver transplantation are required. There is still no specific drug therapy for NAFLD, but TCM shows its advantages in the treatment of this complex metabolic for its holistic concept and differentiation treatment. TCM, especially TCM compounds, has the characteristics of “multicomponents, multitargets, and multipathways”, which also makes it difficult to study. In this review, we summarize the pharmacological actions of active ingredients from frequently used single herbs in TCM compounds. The integrated mechanism of herb combinations are further discussed to explore the comprehensive effects of TCM compounds on NAFLD. Overall, this target-pathway-faceted integrated regulation of TCM makes it more competitive than any other chemical drugs or active ingredients.

However, several defects still exist in TCM treatment. Firstly, The TCM compounds in registered clinical trials are limited, although some are proved to be effective on animal models. Secondly, long term effects of TCM are difficult to evaluate, because the clinical sample size are limited and the long term follow-up studies are deficient. Last but not the least, the mechanism of TCM on NAFLD are not fully elucidated. Clarifying the activities and mechanism of TCM compounds as a an entirety is necessary for the future drug development of TCM in NAFLD. For this purpose, metabolomics, transcriptomics, network pharmacology, 16S rRNA gene sequencing, and other omic analyses are used in TCM studies in recent years. Network pharmacology combined with systemic biology, multi-directional pharmacology, bioinformatics, and other disciplines also open up a new way to study the complex TCM based on the “disease-gene-target-medicine” interaction network. The use of these omics analysis is in accordance with the concept of “holism” and could provide systematic views for future studies on TCM mechanism [20].

## Data Availability

The datasets are included within the article.

## Abbreviations

NAFLD: Nonalcoholic fatty liver disease
TCM: Traditional Chinese Medicine
MAFLD: Metabolic associated fatty liver disease
NAFL: Nonalcoholic fatty liver
NASH: Nonalcoholic steatohepatitis
HF: Hepatic fibrosis
HS: Hepatic sclerosis
HCC: Hepatocellular carcinoma
FXR: Farnesoid X receptor
ASK1: Apoptosis signal-regulating kinase 1
HFD: High fat diet
MCD: Methionine- and choline-deficient
ROS: Reactive oxygen species
LPS: Lipopolysaccharide
HFHFr: High fat and high fructose
LXR: Liver X Receptor
GSH-Px: Glutathione peroxidase
SOD: Superoxide dismutase
CCl_4_: Carbon tetrachloride
TCA cycle: Tricarboxylic acid cycle
PA: Palmitic acid
FFA: Free fatty acid
